# From space group to space groupoid: the partial symmetry of low-temperature *E*-vanillyl oxime

**DOI:** 10.1107/S2052520619008461

**Published:** 2019-07-23

**Authors:** Katharina Ehrmann, Stefan Baudis, Berthold Stöger

**Affiliations:** a Institute of Applied Synthetic Chemistry, TU Wien, Getreidemarkt 9, 1060 Vienna, Austria; b X-ray Centre, TU Wien, 1060 Vienna, Austria

**Keywords:** phase transitions, local symmetry, groupoids, partial symmetry

## Abstract

The phase transition of *E*-vanillyl oxime {1-[(*E*)-(hydroxyimino)methyl]-4-hydroxy-3-methoxybenzene, C_8_H_9_NO_3_} has been analysed by single-crystal and powder X-ray diffraction. The high-temperature (HT) phase (*P*2_1_/*a*, *Z*′ = 1) transforms into the low-temperature (LT) phase (threefold superstructure, , *Z*′ = 6) at *ca* 190 K. The point operations lost on cooling, {*m*_[010]_, 2_[010]_}, are retained as twin operations and constitute the twin law. The screw rotations and glide reflections are retained in the LT phase as partial operations acting on a subset of Euclidean space . The full symmetry of the LT phase, including partial operations, is described by a disconnected space groupoid which is built of three connected components.

## Abstract 2

The partial symmetry of low-temperature *E*-vanillyl oxime is analysed using space groupoids.

## 1. Introduction   

The vanillin-derived oxime 1-[(*E*)-(hydroxyimino)methyl]-4-hydroxy-3-methoxybenzene, **1** [Chem scheme1], is a key precursor in the synthesis of bioactive compounds, notably members of the capsaicinoid family found in hot pepper (Gannett *et al.*, 1988 ▸). The crystal structure of **1** [Chem scheme1] at room temperature has been determined by Jerslev & Larsen (1991 ▸). The authors noted that the crystals feature a phase transition on cooling, but owing to twinning no low-temperature structure was determined with their point-detector-equipped diffractometer system.
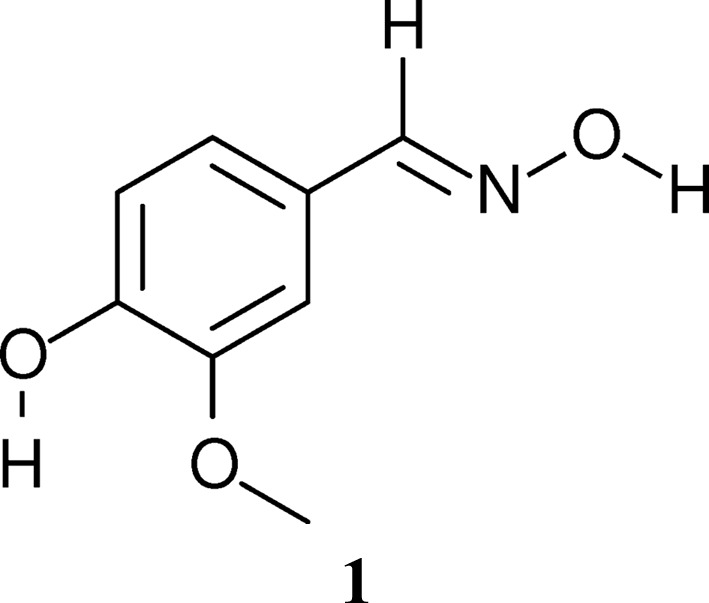


Our group is interested in the symmetry aspects of phase transitions (group/subgroup relations, twinning). Since **1** [Chem scheme1] is easily synthesized from commercial vanillin and hydroxyl­ammonium chloride, we decided to re-evaluate the low-temperature (LT) polymorph using a modern diffractometer system equipped with a two-dimensional detector. Indeed, with such a setup, structural characterization of the twinned LT crystals was unproblematic. In this work, we give a detailed symmetry analysis of the phase transition of **1** [Chem scheme1].

When a crystal structure undergoes a phase transition with a group–subgroup relation, the symmetry operations lost at the transition are retained as operations mapping either orientation (twin) or antiphase domains (in the case of pure translations). Thus, the operations remain active as domain-mapping operations. The phase transition of **1** [Chem scheme1] is of this kind. But here, additionally, some of the lost operations remain not only active as domain-mapping operations, but also inside the unit cell for distinct subspaces of the structure. They thus represent an intermediate step between retained and lost symmetry operations. Special attention will be paid to these partial operations.

## 2. Experimental   

### 2.1. Preparation   

Compound **1** [Chem scheme1] was synthesized by reacting vanillin with hydroxylammonium chloride in MeOH and using K_2_CO_3_ as a base. After cooling and filtration, large (>1 mm edge length) yellow blocks were grown by recrystallization from MeOH.

### 2.2. Single-crystal diffraction   

Intensity data from single crystals of **1** [Chem scheme1] were collected on a Bruker Kappa APEX II diffractometer system equipped with a CCD detector in a dry stream of nitrogen at 300 (high temperature, HT) and 100 K (LT) using graphite-monochromated Mo *K*α radiation. Processing of the HT data was routine. Reflections of the LT data were attributed to two domains using the *RLATT* tool (Bruker, 2017 ▸). Frame data were reduced to intensity values (in the LT case with overlap information) using *SAINT-Plus* (Bruker, 2017 ▸). The structures were solved with *SHELXT* (Sheldrick, 2015 ▸) and refined with *Jana2006 ▸* (Petříček *et al.*, 2014 ▸) against *F* ^2^. Molecular graphics were produced with the program *Mercury* (Macrae *et al.*, 2008 ▸). Electron-density maps were plotted with *MCE* (Husák & Kratochvíl, 2003 ▸). Crystal and instrumental data are summarized in Table 1 ▸.

### 2.3. Settings and labelling   

**Figure 1:**
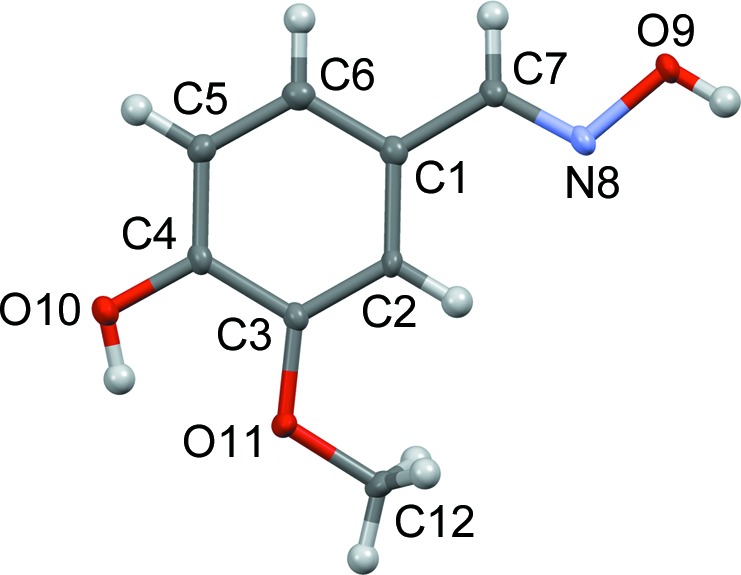
The atom-labelling scheme for **1** [Chem scheme1] according to Jerslev & Larsen (1991 ▸). The *a* molecule of the LT phase is shown, with ellipsoids drawn at the 50% probability level, except for H atoms which are represented as spheres of arbitrary radii. Colour code: C grey, N blue, O red and H white.

Refinements of both phases were performed in the usual settings (conventional monoclinic setting with a less obtuse β angle for the HT data and a reduced setting for the LT data) to minimize correlation of positional and displacement parameters [HT: *P*2_1_/*c*, *a* ≃ 6.37 Å, *b* ≃ 16.65 Å, *c* ≃ 7.58 Å, β ≃ 93.95°; LT: 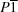: *a* ≃ 7.36 Å, *b* ≃ 17.56 Å, *c* ≃ 19.38 Å, α ≃ 68.69°, β ≃ 83.98°, γ ≃ 87.90°]. For the HT phase, this corresponds to the setting chosen by Jerslev & Larsen (1991 ▸). In this case, the published labelling (Fig. 1 ▸) and atomic coordinates were adopted. For the LT phase, which contains *Z*′ = 6 crystallographically independent molecules, the letters *a*–*f* were appended to disambiguate the atom names.

Unfortunately, the structures are difficult to relate using these settings. Therefore, the structural descriptions will be based on unconventional settings (Nespolo & Aroyo, 2016 ▸) with highly acute and obtuse cell angles. The **a** and **b** basis vectors of these settings span distinct crystallochemical layers extending in the (001) plane and the **c** vector connects two adjacent layers. Moreover, the [100] direction and (010) planes of both structure models are equivalent. These common directions and planes will therefore be given without differentiation. For others, an ‘HT’ or ‘LT’ subscript will specify the appropriate reference system. In the HT phase, the lattices are related by (**a**_HT_, **b**_HT_, **c**_HT_) = (2**a**_HT,C_ + **c**_HT,C_, **b**_HT,C_, −**a**_HT,C_) (*P*2_1_/*a*, *a* ≃ 14.37 Å, *b* ≃ 16.65 Å, *c* ≃ 6.37 Å, β ≃ 148.26°), whereby the subscript ‘C’ stands for ‘conventional’ and indicates the usual setting. In the LT phase, the relationship is more complex and can be expressed by
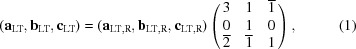
(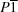, *a* ≃ 42.55 Å, *b* ≃ 21.69 Å, *c* ≃ 20.00 Å, α ≃ 130.36°, β ≃ 170.40°, γ ≃ 48.97°), where ‘R’ stands for ‘reduced’. The positional coordinates and displacement parameters with respect to the unconventional settings are deposited in CIF format in the supporting information. An overview of the cell parameters is compiled in Table 2 ▸.

### 2.4. Displacement parameters   

Some atoms of the HT phase exhibited strongly anisotropic atomic displacement parameters (ADPs) and difference electron-density peaks were observed in the vicinity of these atoms. Attempts to model these positions as occupationally disordered failed (the distinct atoms collapsed to a single position). Therefore, the atoms were described using anharmonic ADPs up to tensor rank 4. With increased rank, the residuals improved distinctly (Table 3 ▸). Positive difference electron-density peaks decreased when increasing the rank to 3, but did not improve further when going to 4. Since rank 4 tensors resulted in a low data-to-parameter ratio of 5.7 and unreasonable geometries of the probability density, the data discussed herein are based on the rank 3 refinements. A short comparison of the refinements is given in the supporting information.

### 2.5. Low-temperature X-ray powder diffraction   

Low-temperature X-ray powder diffraction experiments were performed on a Panalytical X’Pert Pro diffractometer equipped with an Oxford PheniX cryo-chamber in Bragg–Brentano geometry using Cu *K*α_1,2_ radiation (λ = 1.540598 and 1.544426 Å) with an Ni filter and an X’celerator multi-channel detector. The ground bulk sample was placed on an Si single crystal cut along the (711) plane. Scans were recorded in a vacuum in the 2θ = 10–70° range in 5 K steps from 250 to 100 K and back to 250 K with heating and cooling rates of 1 K min^−1^ and 5 min isotherms between scans.

## 3. Results and discussion   

### 3.1. Crystal chemistry   

**Figure 2:**
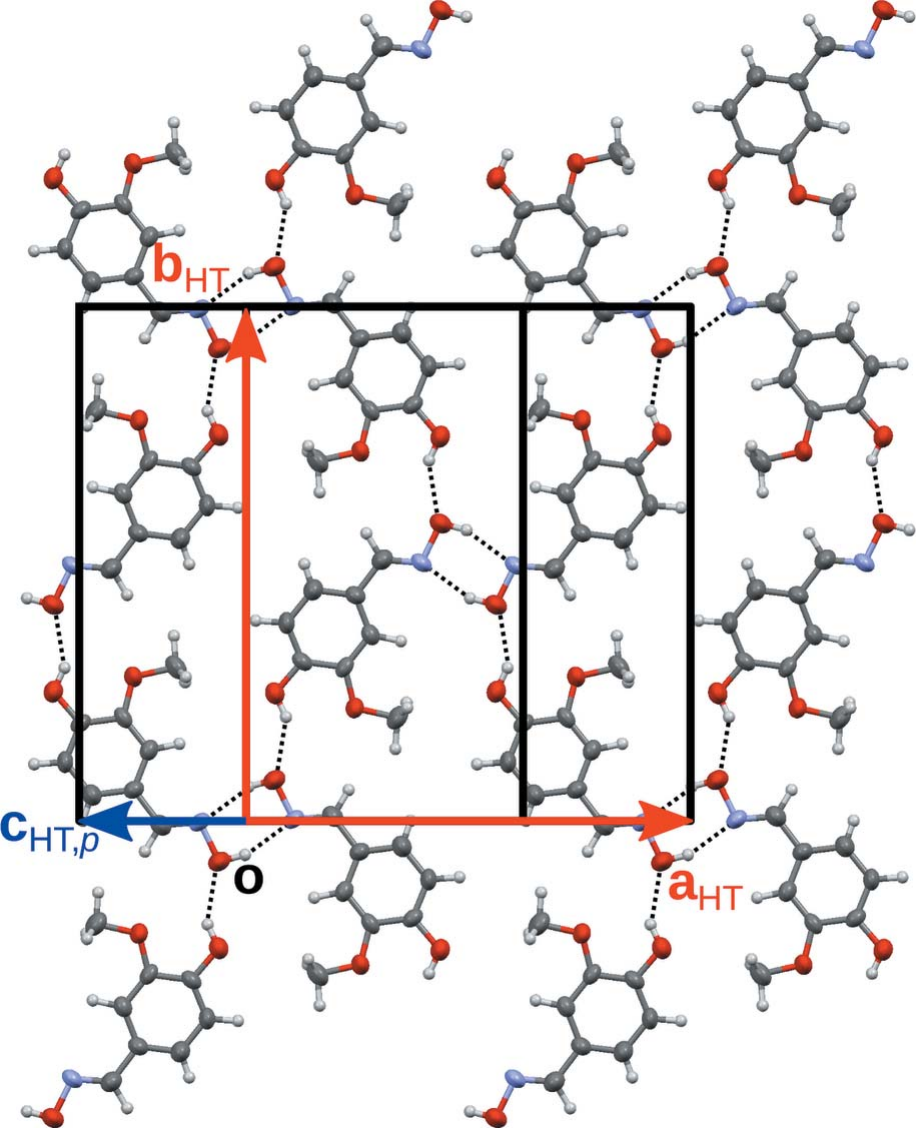
The crystallochemical layer in the HT phase of **1** [Chem scheme1] projected onto the (001) layer plane. Equivalent ADP ellipsoids are drawn at the 50% probability level. Strong hydrogen bonds are represented by dashed lines. The atom colour code is as in Fig. 1 ▸. The unit-cell edges are drawn in black. For clarity, lattice vectors in and out of the drawing plane are drawn in red and blue, respectively.

Compound **1** [Chem scheme1] crystallizes in a structure built of distinct crystallochemical layers parallel to (001) (Fig. 2 ▸). In these layers, the molecules are connected by strong O—H⋯N and O—H⋯O hydrogen bonds, as has already been discussed by Jerslev & Larsen (1991 ▸). The oxime units are connected, forming molecule pairs located on centres of inversion in the HT phase. Moreover, the phenol groups donate to the O atom of the oxime units. Thus, a two-dimensional network composed of hexameric rings is formed. The cycles are symmetric by inversion in the HT phase. The crystallo­chemical layers are connected by van der Waals interactions.

### 3.2. Space-group symmetry reduction and twinning   

The LT phase is a threefold superstructure that can be derived from the HT phase. The basis vectors of both phases are related up to minor distortions by
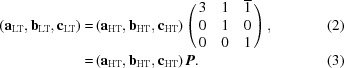
A schematic comparison of the lattices is provided in the supporting information. Accordingly, the reciprocal bases are related by
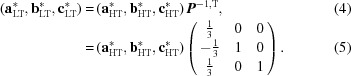
In other words,
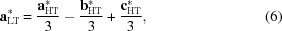
and therefore the LT phase can be considered as a threefold commensurately modulated structure with the modulation wavevector
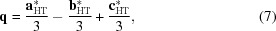
(modulo minor deviations from monoclinic metrics).

Since the lattice of the LT phase is triclinic, the point symmetry of the HT phase cannot be retained. Indeed, it is reduced by an index of 2 from 2/*m* to 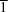. Thus, in total the symmetry groups of the HT and LT phases are related by a group/subgroup relation of index 6, which can be decomposed into two maximal group/subgroup relations according to Hermann’s theorem (Hermann, 1929 ▸):

(i) A *translationengleiche* (same lattice, different crystal class) symmetry reduction of index 2.

(ii) A *klassengleiche* (same crystal class, different lattice) symmetry reduction of index 3.

The lost point operations are retained as twin operations in the LT crystal. Accordingly, the twin law consists of the operations 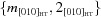. Since the superstructure is threefold, the twin index is 3 (overlap of every third reflection). The twin obliquity, which is derived from the lattice parameters, is 0.28°.

Owing to the threefold superstructure, each of the two twin domains may exist in three antiphase domain states, which are related by the translations of the HT phase that are not symmetry operations of the LT phase. Since domains related by translation produce the same diffraction pattern, these antiphase domains are difficult to observe by diffraction. They might, for example, cause enlargement of reflections owing to a limited domain size. No such effects were observed.

### 3.3. Partial operations in the LT phase   

**Figure 3:**
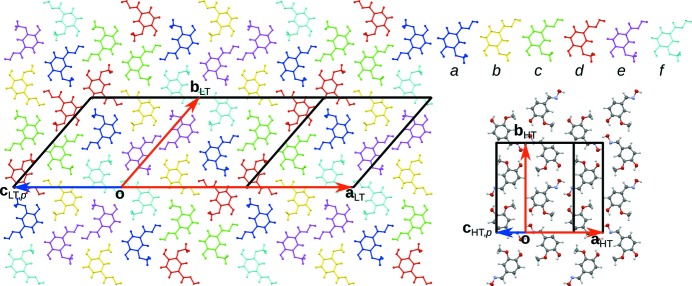
A comparison of a crystallochemical layer (left) in the LT phase and (right) in the HT phase, projected onto the (001) layer plane. The HT-phase atom colours are as in Fig. 1 ▸. The molecules in the LT phase are coloured according to space-group symmetry equivalence.

**Figure 4:**
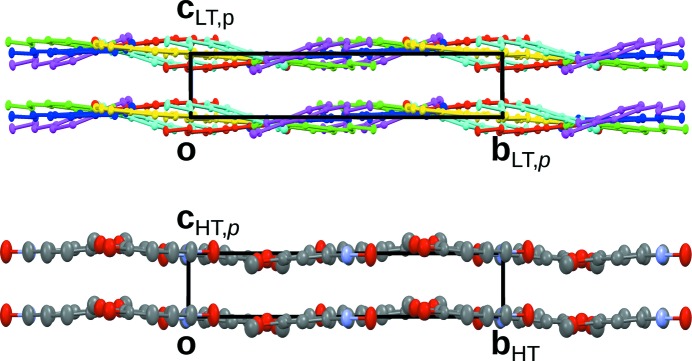
Comparison of (top) the LT phase and (bottom) the HT phase, viewed down [100]. Colours are as in Fig. 3 ▸. H atoms have been omitted for clarity.

Fig. 3 ▸ gives a comparison of the crystallochemical layers of the HT and LT phases projected onto the (001) layer plane. The molecules in the LT phase are coloured according to space-group symmetry equivalence. The LT layer can be considered as a threefold commensurately modulated form of the HT layer with the modulation wavevector
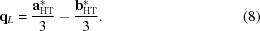
In the [010]_HT_ direction a succession of all six non-equivalent molecules is observed. In the (001) projection, the HT and LT structures are virtually indistinguishable. In projection along [100], on the other hand, the orientations of the molecules in the LT phase exhibit a pronounced deviation from the HT phase (Fig. 4 ▸).

**Figure 5:**
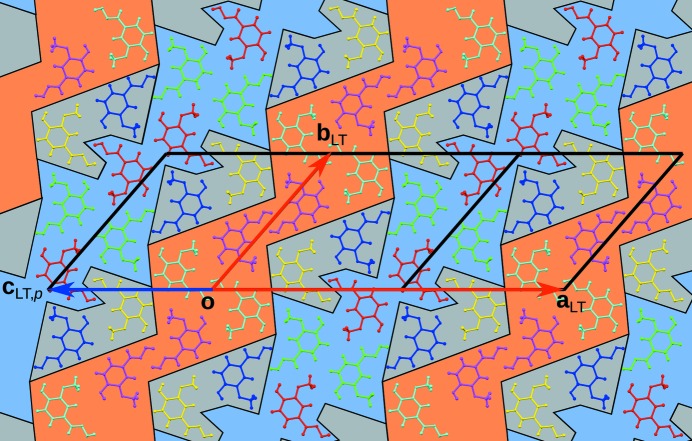
A layer of the LT phase projected onto the (001) layer plane. Molecules are coloured according to space-group symmetry equivalence as in Fig. 3 ▸, and the background is coloured according to partial operations.

A further observation from the [100] projection is that the *a* and *b* molecules in the LT phase adopt an orientation corresponding to the (averaged) position of the molecules in the HT phase. In a sense, this region of the structure is retained on cooling, though devoid of dynamic disorder. It is marked by a grey background in Fig. 5 ▸. Thus, besides inversion and translation, some of the operations of the *P*2_1_/*a* symmetry of the HT phase are still valid for the LT phase, albeit only for a subset of the molecules. These operations are called partial operations, because they act on a subset of Euclidean space (as in partial functions).

The orientations of the remaining four molecules differ distinctly from those in the HT phase (Fig. 4 ▸). Nevertheless, the molecules of the pairs *c*/*d* and *e*/*f* each feature strikingly similar deviations. Indeed the molecules of these pairs are likewise related by a subset of the operations of the HT phase. The resulting regions of molecules equivalent by partial operations are marked by blue (*c*/*d*) and orange (*e*/*f*) backgrounds in Fig. 5 ▸.

### 3.4. Groupoids   

The algebraic structure describing the whole symmetry of a structure, including partial operations, is a space groupoid (Ito & Sadanaga, 1976 ▸). Multiple equivalent definitions of a groupoid have been given. Here, we use groupoids in the categorical sense (Ehresmann, 1957 ▸; Simmons, 2011 ▸). A groupoid 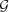 is composed of a set of objects 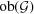 and a set of operations (also called morphisms) 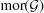. Each operation 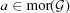 has a source and a target object 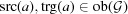.

The fundamental difference between groups and groupoids is that the composition of groupoid operations is not closed. The composition 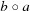 (*b* after *a*) is defined if and only if the target of the first is the source of the second operation [trg(*a*) = src(*b*)]. The source and target of the composed operation are the source of the first and the target of the second operation: 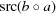 = src(*a*) and 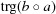 = trg(*b*). In diagram form (operations *a*, *b* and objects *i*, *j*, *k*):
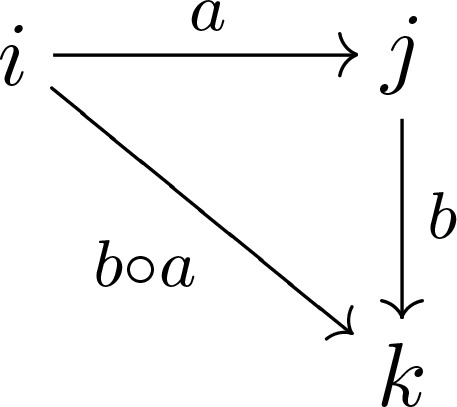


The remaining group axioms remain fulfilled. The composition is associative: 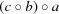 = 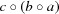. For every object 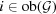 there is a neutral element 1_*i*_ such that 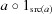 = *a* and 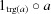 = *a*. For every 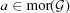 there exists an inverse *a* ^−1^ such that 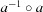 = 1_src(*a*)_ and 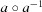 = 1_trg(*a*)_.

In a symmetry description of the LT phase by a groupoid 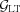, the objects 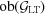 represent the individual molecules. The molecules could, for example, be identified by a unique number. Here, 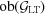 will be the subspace of Euclidean space 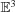 occupied by the individual molecules. 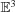 will be partitioned in such a way that every point belongs to at least one molecule or a boundary between two molecules and the space occupied by a particular molecule is simply connected (no holes). Thus, a connection between equivalence according to partial operations and topology can be established.

The operations 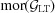 are symmetry operations of the HT phase, associated with a source and a target molecule. Note that here we are not interested in every possible motion relating two molecules, but only those which have a representative in the HT phase. It is easily seen that the composition of these operations forms a groupoid. In the HT phase each molecule is mapped onto every other one by exactly one operation (*Z*′ = 1, molecule on the general position). Thus, in the LT phase two molecules are related by either one or zero partial operations.

Since not all objects 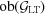 are related by operations 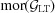 (*e.g. a* and *c* molecules), 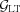 is said to be disconnected. It can be decomposed into three connected components according to 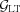 = 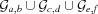, where 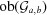 contains only the subspaces of 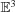 occupied by the *a* and *b* molecules, *etc*.

**Figure 6:**
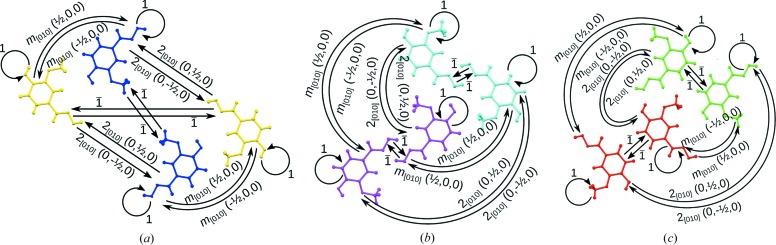
Operations relating (*a*) four *a* and *b* molecules, (*b*) four *c* and *d* molecules and (*c*) four *e* and *f* molecules. Each point operation of the HT phase has a representative. Molecule colours are as in Fig. 3 ▸.

In Fig. 6 ▸, the operations relating a subsection of the mol­ecules are schematized. More precisely, Fig. 6 ▸(*a*) represents a full subgroupoid of 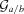: it contains a part of the objects, but all operations between these objects. Figs. 6 ▸(*b*) and 6 ▸(*c*) show full subgroupoids of 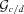 and 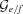, respectively. Notably, each of these subgroupoids has a representative of a point-group operation of the HT phase. In a sense, the point group of the overall point symmetry, including partial operations, is still 2/*m*. Table 4 ▸ lists the groupoid 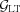 in table form, whereby operations and objects are given up to the translation lattice of the LT phase.

### 3.5. Partial operation equivalence and topology   

By choosing subspaces of 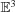 as groupoid objects, a relationship between partial operations and topology is established. All objects of a connected component are related by partial operations. Thus, the areas with grey, blue and orange backgrounds in Fig. 5 ▸ are mapped onto themselves by the operations of the 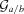, 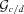 and 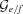 subgroupoids of 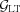, respectively. These regions can be written as the union of groupoid objects, *viz.* as 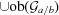, 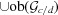 and 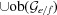.

**Figure 7:**
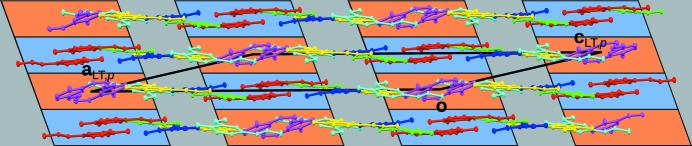
The LT phase viewed down **b**_LT_. Molecules are coloured according to space-group symmetry equivalence, and the background is coloured according to equivalence by partial operations (colour codes as in Fig. 5 ▸).

**Figure 8:**
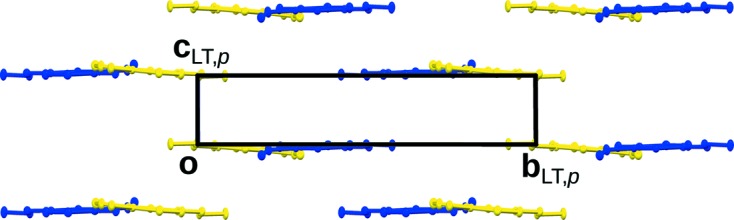
A slab of *a* and *b* molecules viewed down [100]. Molecule colours are as in Fig. 6 ▸.

Fig. 5 ▸ suggests topologies periodic in zero (*a*/*b*) and one (*c*/*d* and *e*/*f*) dimensions, the last extending normal to **q**_*L*_ [equation (8)]. This picture reverses when considering the interlayer van der Waals interactions (Fig. 7 ▸). The *a*/*b* pairs are connected to two-dimensional sheets with holes in a checkerboard pattern (Fig. 8 ▸). The *c*/*d* and *e*/*f* rods, on the other hand, do not connect to other rods of the same kind and therefore remain rods even when considering the full structure (Fig. 7 ▸). These two- and one-dimensionally periodic subspaces extend normal to the modulation vector **q** [equation (7)].

### 3.6. Desymmetrization   

**Figure 9:**
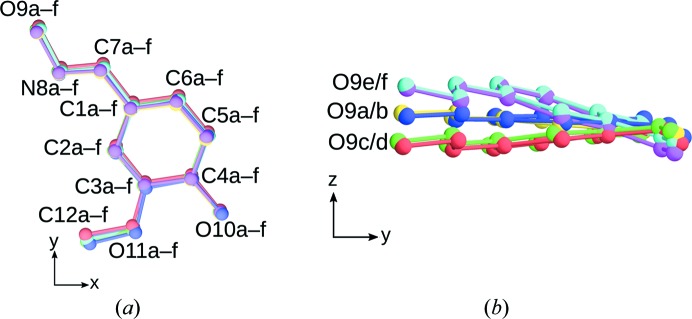
An overlay of the six molecules in the LT phase when mapped onto each other by a symmetry operation of the HT phase projected (*a*) onto (001) and (*b*) down [010]_HT_. Molecule colours are as in Fig. 3 ▸.

Since partial operations are in general only valid for subsets of a crystal, interactions with the remaining parts will usually induce slight deviations from the idealized model. To quantify this desymmetrization, the coordinates of the LT phase were transformed into a pseudo-monoclinic coordinate system corresponding to the HT phase. The cell parameters thus obtained are *a* = 14.182 Å, *b* = 16.361 Å, *c* = 19.995 Å, α = 90.22°, β = 170.40°, γ = 89.82°, showing a slight deviation from the ideal metrics (α = γ = 90°). The cell parameters were then idealized as α = γ = 90°, by projecting **b** onto **c** × **a**. Finally, the symmetry operations of the *P*2_1_/*a* group of the HT phase were applied to map the molecules onto the ‘same’ position. In Fig. 9 ▸, an overlay of the six molecules thus obtained is given. Whereas in projection on (001) all molecules map onto each other (see Section 3.3), in projection along [100] the *a*/*b*, *c*/*d* and *e*/*f* pairs are clearly identified. Table 5 ▸ lists the distances between the corresponding atoms in these pairs. In general the distance between the atoms related by partial operations is small, on average <0.2 Å, demonstrating the validity of the description.

### 3.7. Symmetry reduction and topology   

Traditionally, displacive phase transitions are characterized by group/subgroup relations (Müller, 2013 ▸). But, as has been shown in Section 3.6, on cooling some of the symmetry operations remain active for a subspace of 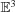. To describe the symmetry relationship using these partial operations, one can consider the space group of the HT phase as being a groupoid 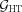 with a single object, *viz.* 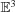. Each operation of 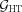 maps 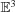 onto itself.


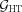
 and 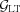 can be related by a groupoid functor (the category theoretical equivalent of a homomorphism) *F*: 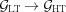. A functor maps the objects and operations of a groupoid, in a way that is compatible with the structure of the groupoids: 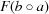 = 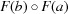. Here, *F* maps every object of 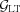 onto the single object of 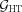 and every partial operation of the LT phase onto the corresponding global operation of the HT phase. This kind of functor is often designated as a ‘forgetful functor’ (Simmons, 2011 ▸), as it ‘forgets’ the structure, in this case the objects (molecules) that the operations relate.

It has to be emphasized that the image of *F* (the operations in 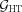 corresponding to operations in 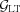) does *not* form a groupoid. For example, double application of the 2_1_ operations of Fig. 6 ▸ produces a lattice translation, which is not in the image of *F*. The preimage *F* ^−1^(*a*) of a global operation 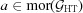 in the HT phase is the set of all corresponding (partial) operations in the LT phase. More interesting than *F* ^−1^(*a*) itself is the set of all source and target objects of the operations in *F* ^−1^(*a*), which will be designated as src(*F* ^−1^(*a*)) and trg(*F* ^−1^(*a*)), respectively.

Three cases can be differentiated:

(i) If 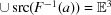, then likewise 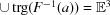 and *a* is a full operation of the LT phase. These correspond to the symmetry operations of the 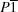 space group of the LT phase.

(ii) If ∪ src(*F* ^−1^(*a*)) = ∅, then likewise ∪ trg(*F* ^−1^(*a*)) = ∅ and the operation is lost on cooling. These are a subset of the translations and inversions.

(iii) If 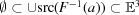, then likewise 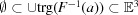. These *a* can be considered as proper partial operations. Examples are the 2_1_ screw rotations and the *a* glide reflections.

Operations of the second and third kind may lead to twinning or anti-phase domains. Thus, twin or anti-phase domain operations may have representatives in the space groupoid. In the case of **1** [Chem scheme1] this is the case for the twin operations but not for the anti-phase domain translations.

**Figure 10:**
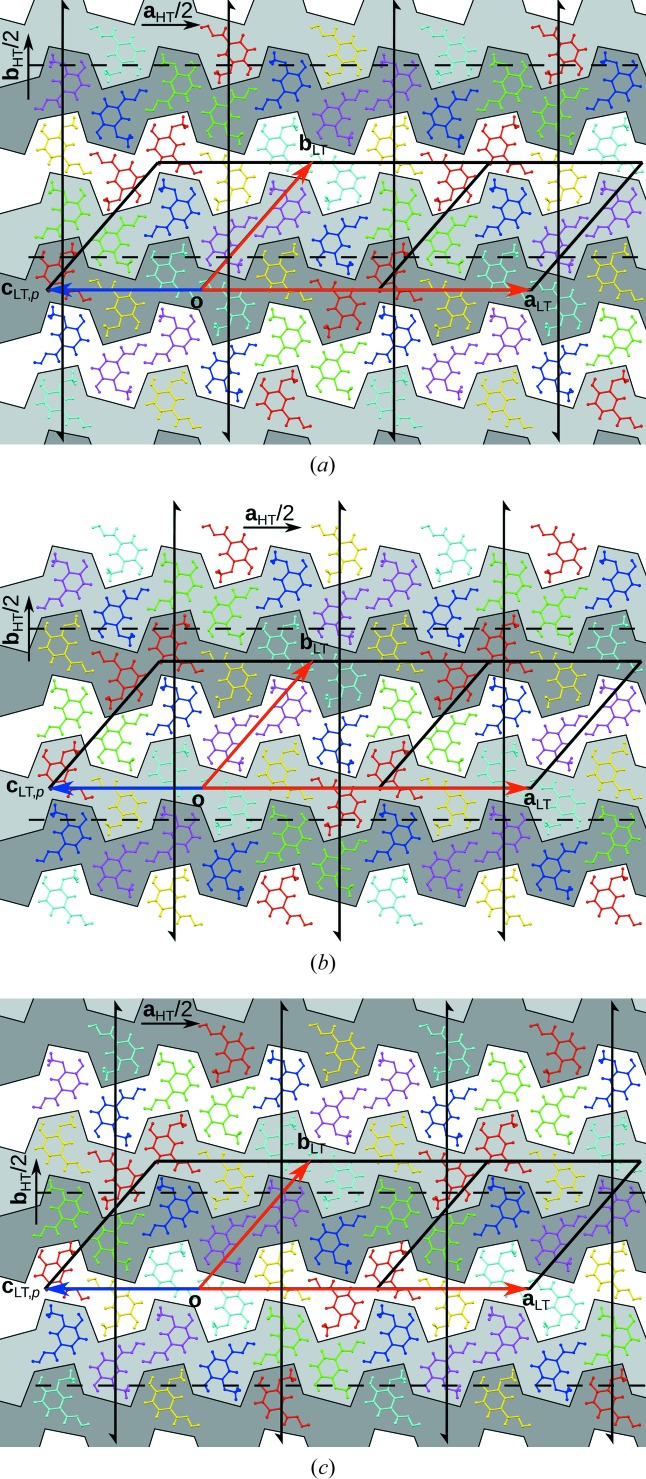
Source (dark grey) and target (light grey) molecules of partial screw rotations and glide planes with intrinsic translation (6*n* + 1)**b**_HT_/2 and (6*n* + 1)**a**_HT_/2, respectively. Molecule colours are as in Fig. 3 ▸.

Fig. 10 ▸ shows the ∪ src(*F* ^−1^(*a*)) (dark grey) and ∪ trg(*F* ^−1^(*a*)) (light grey) regions of screw rotations with intrinsic translation (6*n* + 1)**b**_LT_/2 and glide reflections with intrinsic translation (6*n* + 1)**a**_LT_/2, 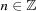. The inverse operations with intrinsic translation (6*n* + 5)**b**_LT_/2 and (6*n* + 5)**a**_LT_/2, 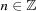 transform the regions marked with light grey into those marked with dark grey. Concerning the source and target objects, there are three kinds of these operations, *viz.* with rotation axes at (¼+3*n*/2, *y*, 0), (¾+3*n*/2, *y*, 0) and (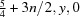) with respect to the basis of the HT phase. The corresponding glide planes are located at (*x*, ¼+3*n*/2, *z*), (*x*, ¾+3*n*/2, *z*) and (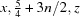) (Fig. 10 ▸).

**Figure 11:**
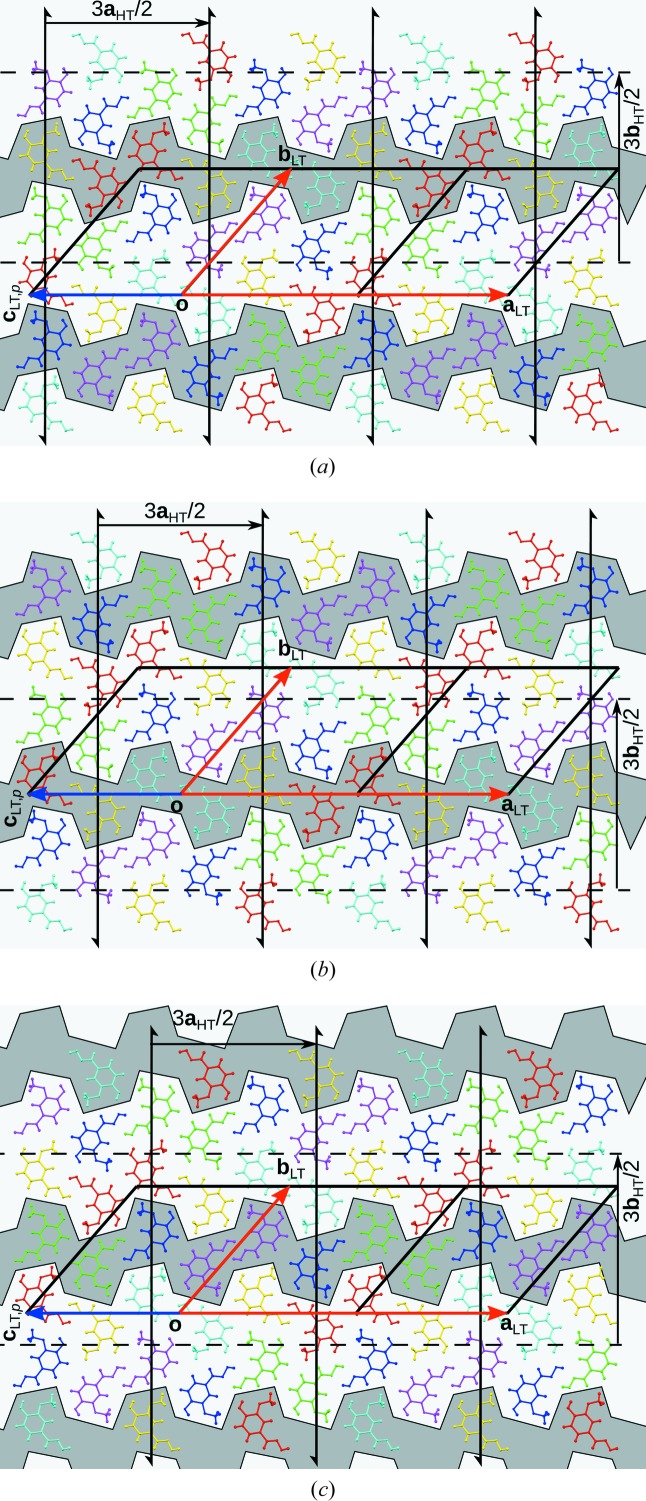
Source and target molecules of partial screw rotations and glide planes with intrinsic translation (6*n* + 3)**b**_LT_/2 and (6*n* + 3)**a**_LT_/2, respectively, in the LT phase. Molecule colours are as in Fig. 3 ▸.

For screw rotations with intrinsic translation (6*n* + 3)**b**_LT_/2 and glide reflections with intrinsic translation (6*n* + 3)**b**_LT_/2, there are again three types of operation. Here, ∪ src(*F* ^−1^(*a*)) = ∪ trg(*F* ^−1^(*a*)) (medium grey in Fig. 11 ▸).

As can be seen in Figs. 10 ▸ and 11 ▸, the partial screws and glides apply to distinct layers parallel to (010). This means that the LT phase can be considered as an order–disorder (OD) structure. Details of such a description are provided in the supporting information.

### 3.8. X-ray powder diffraction   

**Figure 12:**
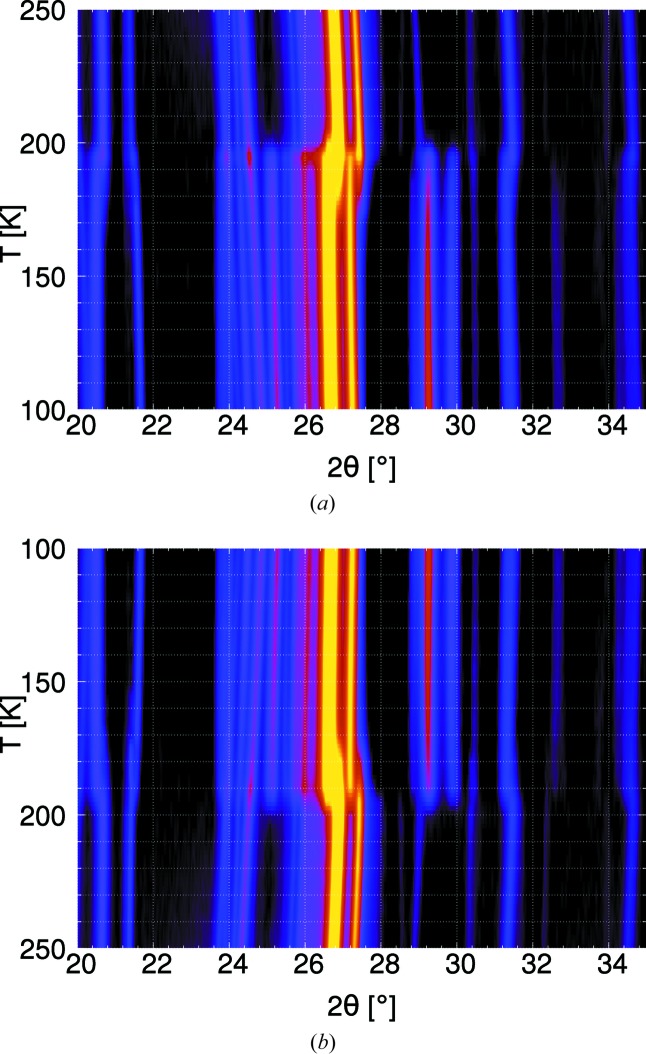
Powder diffraction patterns of **1** (*a*) on heating and (*b*) on cooling, taken at 5 K intervals. Scans are arranged in chronological order from bottom to top.

Since the crystallographic symmetries of the HT and LT phases are related by a non-maximal group/subgroup relationship of index 6, one could suspect intermediate phases. During an attempt to show such a phase by single-crystal diffraction, a crystal spontaneously converted from the HT to the LT phase during data collection at 200 K. To give further proof of the absence of an intermediate phase, low-temperature powder diffraction was performed in a cooling (250→100 K) and a subsequent heating (100→250 K) cycle (Fig. 12 ▸). As expected, neither on heating nor on cooling were additional phases observed. In scans at 190 K (cooling) and 195 K (heating) both phases exist, suggesting a phase transition of the first order.

## 4. Conclusions   

By considering partial operations, we have established a connection between symmetry and topology in two ways, firstly by partitioning 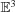 into regions equivalent according to partial operations, and secondly by generalizing the symmetry reduction. A full operation of the HT phase is split into several partial operations in the LT phase, each acting on different subspaces of 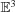. These might be split further in a subsequent hypothetical phase transition. Thus, the concept of symmetry reduction is generalized by considering the subspace of 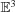 for which an operation of the high-symmetry phase stays active. Previously, only the extreme cases (loss of symmetry, active on ∅ and retention of symmetry, active on 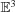) were considered.

More work is necessary to investigate whether other phase transitions can be viewed in the light of partial symmetry. Moreover, the connection between the topologies presented here and the superspace approach of modulated structures is as yet unclear.

## 5. Related literature   

References cited in the supporting information include: Dornberger & Schiff (1961 ▸) and Fichtner (1979 ▸).

## Supplementary Material

Crystal structure: contains datablock(s) ht_r2_conv, ht_r3_conv, ht_r4_conv, lt_conv, ht_r2_unconv, ht_r3_unconv, ht_r4_unconv, lt_unconv. DOI: 10.1107/S2052520619008461/um5028sup1.cif

Structure factors: contains datablock(s) ht_r2_conv. DOI: 10.1107/S2052520619008461/um5028ht_r2_convsup2.hkl

Structure factors: contains datablock(s) ht_r3_conv. DOI: 10.1107/S2052520619008461/um5028ht_r3_convsup3.hkl

Structure factors: contains datablock(s) ht_r4_conv. DOI: 10.1107/S2052520619008461/um5028ht_r4_convsup4.hkl

Structure factors: contains datablock(s) lt_conv. DOI: 10.1107/S2052520619008461/um5028lt_convsup5.hkl

Additional theory. DOI: 10.1107/S2052520619008461/um5028sup6.pdf

CCDC references: 1923015, 1923016, 1923017, 1923018

## Figures and Tables

**Table 1:** Crystal and instrumental data from the single-crystal experiments

	HT phase	LT phase
Chemical formula	C_8_H_9_NO_3_	C_8_H_9_NO_3_
*M*_r_	167.2	167.2
*T* (K)	300	100
θ range (°)	2.45–30.11	1.13–32.75
Radiation	Mo *K*α	Mo *K*α
Crystal description, colour	Block, yellow	Block, yellow
Crystal size (mm)	0.32 × 0.45 × 0.50	0.25 × 0.34 × 0.53
Data collection		
Diffractometer	Bruker Kappa APEX II	Bruker Kappa APEX II
Absorption correction	Multi-scan (*SADABS*)	Multi-scan (*TWINABS*)
*T*_min_, *T*_max_	0.97, 0.97	0.94, 0.97
Data reduction		
Reflections measured, unique, *F* ^2^ > 3σ(*F* ^2^)	8632, 2358, 1540	79 437, 16 868, 13 835
*R*_int_	0.0217	0.0461

**Table 2:** Overview of the conventional and non-conventional cells

	HT phase		LT phase	
	Conventional	Unconventional	Conventional	Unconventional
Space group	*P*2_1_/*c*	*P*2_1_/*c*	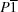	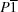
*a* (Å)	6.3704 (3)	14.3673 (6)	7.3634 (8)	42.550 (4)
*b* (Å)	16.6534 (9)	16.6534 (9)	17.5614 (18)	21.687 (2)
*c* (Å)	7.5759 (4)	6.3704 (3)	19.3782 (19)	19.9953 (19)
α (°)	90	90	68.688 (3)	130.368 (12)
β (°)	93.9516 (16)	148.261 (7)	83.981 (3)	170.40 (4)
γ (°)	90	90	87.900 (3)	48.973 (11)
*V* (Å^3^)	801.81 (7)	801.81 (18)	2321.6 (4)	2322 (11)
*Z*, *Z*′	4, 1	4, 1	12, 6	12, 6
Twin operation			*m*_(010)_	*m*_(010)_
Twin volume ratio			52.29:47.71 (8)	52.29:47.71 (8)

**Table 3:** Refinement data for the HT and LT phases, the former with ADP tensors of rank 2 to 4

	HT phase			LT phase
Rank of ADP tensor	2	3	4	2
*R* [*F* ^2^ > 3σ(*F* ^2^)]	0.0471	0.0372	0.0313	0.0411
*wR* [*F* ^2^ > 3σ(*F* ^2^)]	0.1319	0.1031	0.0673	0.1145
*R* (all)	0.0741	0.0641	0.0585	0.0555
*wR* (all)	0.1436	0.1167	0.0762	0.1247
*S*	1.96	1.63	1.73	1.42
No. of parameters	117	237	417	698
Data-to-parameter ratio	20.2	9.9	5.7	24.2
Δρ_max_, Δρ_min_ (e Å^−3^)	−0.20, 0.24	−0.16, 0.16	−0.12, 0.17	−0.24, 0.41

**Table 4:** The groupoid 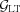 in table form Only one table is shown, as the table applies to all three connected components. Intrinsic translations and positions of geometric elements are given with respect to the basis of the HT phase. Molecules and operations are given modulo the translation lattice of the LT phase. Molecules obtained by a 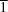 operation of the LT phase are marked with a prime.

	*b*/*d*/*f*	*b*′/*d*′/*f*′	*a*/*c*/*e*	*a*′/*c*′/*e*′
*b*/*d*/*f*	1	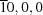	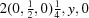	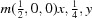
*b*′/*d*′/*f*′	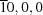	1	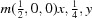	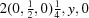
*a*/*c*/*e*	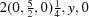	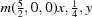	1	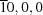
*a*′/*c*′/*e*′	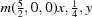	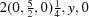	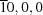	1

**Table 5:** Distances between corresponding atoms in the *a*/*b*, *c*/*d* and *e*/*f* pairs in the LT phase when mapped onto each other by a symmetry operation of the HT phase

	Distances (Å)		
Atom	*a*/*b*	*c*/*d*	*e*/*f*
C1	0.086	0.189	0.098
C2	0.085	0.217	0.156
C3	0.137	0.146	0.185
C4	0.159	0.110	0.134
C5	0.095	0.113	0.089
C6	0.065	0.145	0.073
C7	0.113	0.188	0.074
N8	0.120	0.184	0.064
O9	0.143	0.169	0.048
O10	0.251	0.127	0.130
O11	0.212	0.150	0.265
C12	0.167	0.345	0.245
∅	0.136	0.174	0.130
