# Supplementary material for: From space group to space groupoid: the partial symmetry of low-temperature E-vanillyl oxime
Source: Acta Crystallogr B Struct Sci Cryst Eng Mater. 2019 Jul 23;75(Pt 4):733–41. doi: 10.1107/S2052520619008461 (PMC6690132; doi:10.1107/S2052520619008461)
Supplement: Supplementary file 6 [file b-75-00733-sup6.pdf]

STRUCTURAL SCIENCE
CRYSTAL ENGINEERING
MATERIALS

Volume 75 (2019)

Supporting information for article:

From space group to space groupoid: the partial symmetry of low-temperature *E*-vanillyl-oxime

Katharina Ehrmann, Stefan Baudis and Berthold Stöger

Acta Crystallographica Section B
Structural Science,
Crystal Engineering
and Materials
ISSN 0108-7681

From space group to space groupoid: the partial symmetry of low-temperature E-vanillyl-oxime — Supporting Information

Katharina Ehrmann,[a] Stefan Baudis[a] and Berthold Stöger [b]*

[a]TU Wien, Institute of Applied Synthetic Chemistry, Getreidemarkt 9, 1060 Vienna Austria,
and [b]TU Wien, X-Ray Centre, 1060 Vienna Austria. Correspondence e-mail: bstoeger@mail.tuwien.ac.at

© 0000 International Union of Crystallography

1. Lattice relationships

Fig. S1 shows the relationship of the lattices of the HT- and LT-phases of **1**.

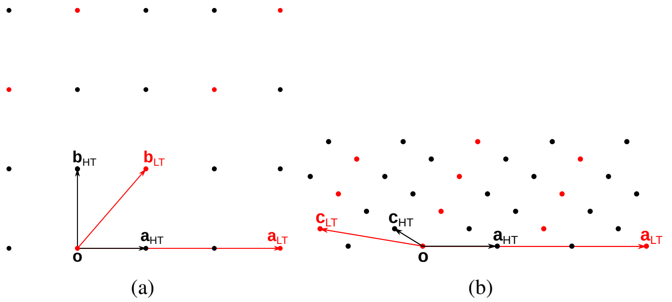

Figure S1
Relationship of the lattices of the HT- and LT-phases exemplified at the (a) $z = 0$ and (b) $y = 0$ planes. Red dots represent nodes of both phases, black dots only of the HT-phase.

2. OD interpretation

The LT-phase can be described as stacking of layers which are related by pseudo-symmetry (Fig. S2). In this light, it is as a classical order-disorder (OD) structure (Dornberger-Schiff & Grell-Niemann, 1961). The OD theory was developed to explain the frequent occurrence of polytypism, though not all polytypes are of the OD type.

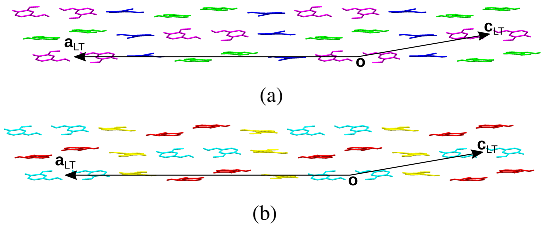

Figure S2
Layers related by partial operations in the LT-phase parallel to (010) projected on the layer plane.

OD structures are likewise described by groupoids, though here the objects are OD layers. All groupoids of OD structures built according to the same *symmetry principle* (Fichtner, 1979) belong to an OD groupoid family, which plays the role of space group types in OD theory. The symbol for the OD groupoid family of the LT-phase reads as

$$\begin{array}{ccccc} P & \bar{1} & (\bar{1}) & \bar{1} & \\ \{ & - & (2_2/n_{r,s}) & - & \} \end{array}$$

According to the first line, the structure is built of A_i layers (i is a sequential number) with $P\bar{1}(\bar{1})\bar{1}$ symmetry, whereby the parentheses indicate the direction lacking translation, and the lattice basis $(\mathbf{a}_{LT}, \mathbf{c}_{LT}) = (3\mathbf{a}_{HT}, -\mathbf{a}_{HT} - \mathbf{c}_{HT})$ (Fig. S2). These symmetry operations are called λ-POs, where λ stands for *layer*. The second line of the symbol indicates operations that relate adjacent layers using generalized Hermann-Mauguin symbols. These operations are called σ-POs, where σ stands for *space*, as these operations map different parts of space. 2_2 is the symbol of a screw rotation with the screw vector $\mathbf{b}_0 = \mathbf{b}_{HT}$ and $n_{r,s}$ of a glide reflection with the glide vector $r\mathbf{c}_{LT}/2 + s\mathbf{a}_{LT}/2$.

The OD character of the structure is due to $r, s \neq \mathbb{Z}$. This means that a $n_{r,s}$ and a $n_{-r,-s}$ σ-PO applied to A_n give two *different* positions of A_{n+1}, respectively. Nevertheless, both (A_n, A_{n+1}) pairs of layers are equivalent. Thus, an infinity of polytypes can be generated by subsequent application of $n_{r,s}$ or $n_{-r,-s}$ σ-POs, respectively. Disregarding desymmetrization and interactions beyond one layer width, these polytypes are locally equivalent and therefore also energetically equivalent.

Two of these polytype are of a maximum degree of order (MDO), meaning that they cannot be decomposed into fragments of simpler polytypes:

- MDO_1 ($P\bar{1}$, $\mathbf{b} = \mathbf{b}_0 + r\mathbf{c}_{LT}/2 + s\mathbf{a}_{LT}/2$): generated by continuous application of $n_{r,s}$.
- MDO_2 ($P2_1$, $\mathbf{b} = 2\mathbf{b}_0$): generated by alternating application of $n_{r,s}$ and $n_{-r,-s}$.

The LT-phase corresponds to the MDO_1 polytype. In a classical OD interpretation if twinning, the twin interface would be regarded as a fragment of the MDO_2 polytype. Here, this interpretation is not compelling, as the LT-phase is derived from a higher-symmetry HT-phase and was not grown layer-by-layer.

The OD description is complementary to the groupoid description above. It is more general, as it describes an infinity of poly-

types. On the flip side, it does not shed light on the observed symmetry reduction from the HT- to the LT-phase.

3. ADPs in the HT-phase

Fig. S3 shows the ADP ellipsoids of the atoms in the HT-phase when modeling with second-rank (harmonic) tensors. Atomic positions that deviate more from their average positions in the LT-phase possess larger displacements in the HT-phase. Moreover, the displacements in the HT-phase are in the direction of the displacements of the LT-phase. One can thus presume that in the HT-phase the molecules dynamically oscillate between the positions of the LT-phase.

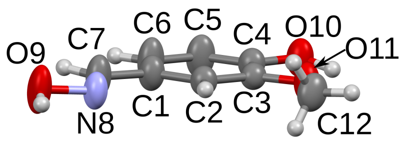

Figure S3

Molecule in the LT-phase from data refined with rank 2 ADP tensors. Ellipsoids are drawn at the 50% probability levels.

Since harmonic ADPs did not satisfactorily describe the dynamic displacements, refinements using higher-order ADP tensors were performed. Electron density of the resulting models are exemplified on the oxime group in Fig. S4. Rank 4 tensors result in unphysical bulges and unexpected splitting of the probability density and were therefore dismissed. The rank 3 tensor model features a distinct deviation from the ellipsoidal shape of the probability density functions. Nevertheless, no splitting corresponding to the atoms in the LT-phase could be evidenced using the present data (resolution limit $\sin\theta/\lambda = 0.71$ Å^{-1}).

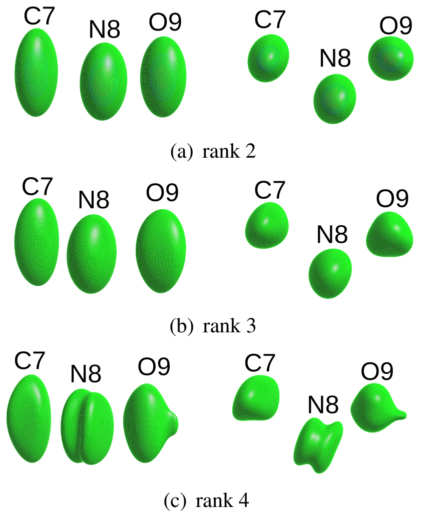

Figure S4

Probability density functions of the atoms of the oxime group in the LT-phase refined with tensors of rank (a) 2, (b) 3 and (c) 4, viewed approximately parallel (left) and perpendicular (right) to the molecular plane. Green lines represent the 0.04 e^- Å^{-3} iso surface.

It has to be noted that, particularly in organic compounds, higher-order ADPs may also model electron density of valence electrons, which are disregarded in a classical refinement. Refinements of the aspherical electron distribution using multipole models did indeed distinctly decrease difference electron density located on bonds. As opposed to the higher-order ADP models, these refinements did not improve reliability factors though.

References

Dornberger-Schiff, K. & Grell-Niemann, H. (1957). *Acta Cryst.*, **14**, 167–177.

Fichtner, K. (1979). *Krist. Techn.*, **9**, 1073–1078.
